# Neoadjuvant serplulimab combined with chemotherapy in locally advanced driver gene-negative squamous NSCLC: a multicenter observational study

**DOI:** 10.3389/fimmu.2026.1882282

**Published:** 2026-09-24

**Authors:** Nanlong Lin, Zeru Yang, Fengqiang Yu, Jianbo Lin

**Affiliations:** 1 Department of Thoracic Surgery, First Affiliated Hospital of Fujian Medical University, Fuzhou, China; 2 Department of Thoracic Surgery, National Regional Medical Center, Binhai Campus of the First Affiliated Hospital, Fujian Medical University, Fuzhou, China; 3 Fujian Medical University, Fuzhou, China

**Keywords:** combine therapy, locally advanced sq-NSCLC, major pathological response, neoadjuvant therapy, serplulimab

## Abstract

**Background:**

Previous evidence demonstrated that neoadjuvant chemo-immunotherapy provides significant prognostic benefits for non-small cell lung cancer (NSCLC). While the efficacy of serplulimab, an anti-PD-1 monoclonal antibody, has been well established in advanced driver gene-negative NSCLC in the ASTRUM-004 trial, its efficacy in the neoadjuvant setting remains unclear. This real-world study aims to evaluate the efficacy and safety of neoadjuvant serplulimab plus chemotherapy in patients with locally advanced squamous NSCLC, with a particular focus on pathological response outcomes.

**Methods:**

This was a multicenter, retrospective–prospective, real-world observational study. Electronic medical records were retrospectively reviewed to identify eligible patients with locally advanced squamous NSCLC who received at least 1 cycle of neoadjuvant serplulimab (300 mg, Q3W) combined with platinum–taxane chemotherapy. Prospective follow-up was conducted to complete courses and outcomes. The primary endpoint was major pathologic response (MPR), and the secondary endpoints included pathological complete response (pCR), objective response rate (ORR), event-free survival (EFS), overall survival (OS), and safety.

**Results:**

Thirty patients were included (median age 64 years; 96.7% male; 86.7% stage III). Most patients received two cycles of neoadjuvant therapy (46.67%), and 80% achieved a radiologic ORR (95% CI, 61.4–92.3%). Surgery was performed on 28 patients (93.33%), with MPR and pCR achieved in 57.1% (95% CI, 39.1–73.5%) and 28.6% (95% CI, 15.3–47.1%), respectively, and an R0 resection rate of 92.9%. Compared with the historical chemotherapy group, the observed MPR was significantly higher (57.1% vs 8.9% and 11.0%; *P* < 0.001). The median EFS reached 29.97 months, and the 12-month EFS rate was 91.8% (95% CI, 81.4–100%). The OS data were immature. Grade 3 adverse events occurred in 6.7% of patients, and no grade 4–5 events or perioperative immune-related complications were reported. Exploratory analyses revealed that a lower post-treatment neutrophil-to-lymphocyte ratio (NLR< 3) was positively correlated with MPR, whereas advanced T stage (T3–T4) was negatively associated.

**Conclusion:**

Neoadjuvant serplulimab-chemotherapy demonstrated promising efficacy and manageable toxicity in real-world locally advanced NSCLC. Survival outcomes require longer follow-up to assess clinical benefit.

## 1 Introduction

Despite decades of multidisciplinary efforts that have substantially improved the incidence and prognosis of non–small cell lung cancer (NSCLC), it still ranks among the most burdensome malignancies worldwide, with an estimated 2.48 million cases in 2022, of which nearly 40–45% occur in China (1, 2). The implementation of population screening programs has facilitated earlier detection, and epidemiological studies indicate that approximately 30% of patients are diagnosed with resectable disease, offering a potential for cure (3–5). However, surgery alone addresses the primary tumor, while the efficacy of perioperative systemic management largely determines recurrence and long-term survival. By enhancing antigen presentation, reenergizing systemic immunity, and providing a durable “tail effect” on survival, PD-1/PD-L1 targeted immunotherapy has clear mechanistic advantages (6, 7). Validated by multiple landmark clinical trials, it has rapidly become a pivotal strategy for the perioperative management of solid tumors, particularly NSCLC in the neoadjuvant setting (8, 9). Notably, compared with adenocarcinoma subtypes of NSCLC, squamous cell carcinoma (SCC) harbors far fewer actionable driver mutations but exhibits greater neoantigenicity and higher levels of immune infiltration (10). These clinicopathological characteristics underscore the biological suitability of squamous NSCLC (sq-NSCLC) for immunotherapy-based treatment strategies (11).

The Phase III trial KEYNOTE-671 demonstrated that perioperative immunochemotherapy significantly improved pathological complete response (pCR; 18.1% vs 4.0%) and event-free survival (EFS; 47.2 months vs 18.3 months, HR = 0.59; 95% CI, 0.48–0.72) compared to chemotherapy alone. Importantly, the benefit appeared even more pronounced in the SCC population, with an EFS HR of 0.51 (95% CI, 0.38–0.69) (12). Additionally, the Phase III ASTRUM-004 trial specifically focused on the population with SCC. The serplulimab group showed significant clinical benefits, including a significant increase in progression-free survival (PFS; median 8.3 vs 5.7 months; HR = 0.53) and an improved objective response rate (ORR; 60.1% vs 40.2%) compared to chemotherapy alone (13). Nevertheless, while these results highlight the potential of serplulimab in advanced sq-NSCLC, evidence regarding its use in the perioperative setting remains lacking. This gap is particularly relevant given the pressing clinical need for effective strategies in resectable squamous disease, where immunotherapy may provide the greatest benefit.

Therefore, we conducted a two-center observational study aimed to evaluate the efficacy and safety of serplulimab in patients with driver gene-negative sq-NSCLC and exploring the feasibility in real-word setting. In addition, a one-sample threshold test was applied to compare MPR with historical data, providing real-world evidence to support future prospective studies and clinical decision-making.

## 2 Methods

### 2.1 Study design and patients

This was a multicenter, retrospective–prospective, real-world observational study conducted at Huashan Hospital, Fudan University and The First Affiliated Hospital of Fujian Medical University in China. Electronic medical records from June 1, 2023, to May 31, 2024, were reviewed to identify patients with locally advanced sq-NSCLC who received at least 1 cycle of neoadjuvant serplulimab (300 mg, Q3W) combined with platinum–taxane chemotherapy. Prospective follow-up from June 1, 2024, to May 31, 2025, was used to complete treatment courses, surgical outcomes, and survival data.

Eligible patients were over 18 years old with histologically confirmed sq-NSCLC, clinical stage I–III per the 8th AJCC edition and deemed resectable or potentially resectable by a multidisciplinary team (MDT). All had no actionable genomic alterations on next-generation sequencing (NGS) covering at least EGFR classical mutations, ALK, ROS1, RET fusions, BRAF V600E, METex14, ERBB2/HER2ex20, and NTRK1/2/3 alterations. Patients required both documented baseline and post-neoadjuvant imaging evaluable by RECIST v1.1, and at least one available clinical outcome, including perioperative, pathological, or survival data in the medical records. Exclusion criteria included prior systemic therapy, palliative-intent treatment, unresectable or metastatic disease, active malignancy within 5 years (except cured skin or *in situ* carcinoma), or critical missing data.

This study was approved by the Ethics Committee of The First Affiliated Hospital of Fujian Medical University (Approval No. MRCTA, ECFAH of FMU [2025]913). All procedures were conducted in accordance with the ethical standards of the institutional research committees and with the principles of the Declaration of Helsinki. Patient data were anonymized prior to analysis to protect confidentiality.

### 2.2 Data collection and outcome assessment

Clinical data collected included demographic characteristics, ECOG status, comorbidities, tumor stage, treatment regimens, surgical details, and pathological findings. Pre- and post-neoadjuvant neutrophil-to-lymphocyte ratio (NLR) values were calculated from routine complete blood count results obtained within 7 days before the first treatment cycle and before surgery, respectively.

Follow-up information was obtained from the medical records, postoperative visits, and telephone calls. The primary endpoint was major pathological response (MPR). The secondary endpoints were pCR, ORR, EFS, OS, and safety. The MPR was defined as ≤10% residual viable tumor cells, and pCR as the absence of viable tumor cells in both primary and nodal sites. ORR was defined as the proportion of patients achieving complete or partial response after neoadjuvant therapy. EFS was measured from treatment initiation to disease progression, recurrence, or death from any cause, whichever occurred first. OS was defined as the duration from the start of treatment to death from any cause. Tumor downstaging was determined by comparing pathological and clinical stages. Safety outcomes were gathered for all patients, with severity classified according to CTCAE version 5.0.

### 2.3 Statistical analysis

All analyses were performed using R software (version 4.4.3). Continuous variables were tested for normality using the Shapiro–Wilk test. Normally distributed variables were presented as mean ± SD and compared with the independent-samples *t*-test. Non-normally distributed variables were summarized as median (range) and compared using the Mann–Whitney *U* test or Kruskal–Wallis *H* test. Categorical variables were expressed as counts (N) and percentages (%), and group comparisons were conducted using Fisher’s exact test. Survival outcomes were estimated with the Kaplan–Meier method, and 95% confidence intervals (CIs) were reported. An exact one-sample threshold (one-sided) test was used to compare the observed MPR with historical benchmarks for neoadjuvant chemotherapy, with α = 0.05. Historical reference values were taken from (14, 15). To explore factors associated with MPR, univariate logistic regression was performed to estimate odds ratios (ORs) and 95% CIs. All other statistical tests were two-sided, with a significance level of α = 0.05, except for the one-sample threshold test, which was one-sided. *P* < 0.05 was considered statistically significant. Sample size was calculated using PASS 2021 software based on a one-sample exact binomial test (one-sided α = 0.05, power = 80%). With a null hypothesis MPR rate of 8.9% (based on the chemotherapy arm of CheckMate-816) and an expected MPR rate of 25% under the alternative hypothesis, a minimum of 27 patients was required. Accounting for a potential dropout rate of approximately 10%, a target enrollment of 30 patients was planned.

Three analysis sets were established for efficacy and survival assessment. The Full Analysis Set (FAS) included all patients (n = 30). As supportive analyses, the Per-Protocol Set (PPS) consisted of patients who underwent surgery with available pathological assessment (n = 28), allowing the estimation of treatment efficacy among patients who actually received definitive surgical management. In addition, the sensitivity analysis Set A comprised patients with clinical stage II–III disease who completed neoadjuvant therapy and had surgery within 8 weeks (n = 21). This set was designed to reflect a typical resectable population treated within the surgical timelines commonly observed in clinical practice, thereby assessing the robustness and representativeness of the primary findings.

## 3 Results

### 3.1 Baseline characteristics

A total of 30 eligible patients were included in the analysis. The baseline characteristics of study patients are summarized in **Table 1**. The median age was 64 years (range, 54–81). Most patients were male (96.7%), and all patients had an ECOG performance status of 0. Twelve patients (40.00%) were aged ≥65 years, and 36.67% had a history of smoking. Six patients (20.00%) had comorbidities, including hypertension and diabetes. Regarding tumor characteristics, most patients had stage III disease (86.67%), indicating a high baseline disease burden, with 46.67% of tumors being T3–T4 stage, and 43.3% presenting with clinical N2 involvement. PD-L1 tumor proportion score (TPS) was <1% in 76.67% of patients. There were no statistically significant differences in baseline characteristics between the MPR and non-MPR groups (all *P* > 0.05).

**Table 1:** Baseline characteristics of included patients. .

Characteristic	Overall (n = 30)*	MPR (n = 16)	Non-MPR (n = 12)	*P* value
Age (years), median(range)	64.00 (54–81)	66 (56–81)	64.00 (54–72)	0.279
Age (years), n (%)				0.136
<65	18(60.00)	7(43.75)	9(75.00)	
≥65	12 (40.00)	9 (56.25)	3 (25.00)	
Gender, n (%)				1.000
Male	29 (96.67)	15 (93.75)	12 (100.00)	
Female	1 (3.33)	1 (6.25)	0 (0.00)	
Smoking history, n (%)				1.000
Yes	11 (36.67)	6 (37.05)	4 (33.33)	
Current	5 (16.67)	1 (6.25)	3 (25.00)	
No	19 (63.33)	10 (62.50)	8 (66.67)	
ECOG, n (%)				
0	30 (100.00)	16 (100.00)	12 (100.00)	
Comorbidity^#^, n (%)	6 (20.00)	2 (12.50)	3 (25.00)	
Yes	6(20.00)	2(12.50)	3(25.00)	
Clinical T stage, n (%)				0.053
T1-T2	16 (53.33)	12 (75.00)	4 (33.33)	
T3-T4	14 (46.67)	4 (25.00)	8 (66.67)	
Clinical N stage, n (%)				1.000
N0	4 (13.33)	2 (12.50)	1 (8.33)	
N+	26 (86.67)	14 (87.50)	11 (91.67)	
N2	13 (43.33)	6 (37.50)	7 (58.33)	
Clinical stage, n (%)				0.238
IB	1 (3.33)	1 (6.25)	0 (0.00)	
II	3 (10.00)	3 (18.75)	0 (0.00)	
III	26 (86.67)	12 (75.00)	12 (100.00)	
PD-L1 TPS, n (%)				1.000
<1%	23 (76.67)	12 (75.00)	9 (75.00)	
1-49%	4 (13.33)	2 (12.50)	2 (16.67)	
≥50%	3 (10.00)	2 (12.50)	1 (8.33)	
Follow up Time, median (range)	8.89 (0.10,29.97)	6.24 (2.63,23.10)	10.47 (4.77,24.67)	

*One patient has not yet undergone surgery, and one patient had missing surgical information.

^#^Comorbidities included hypertension and diabetes mellitus.

### 3.2 Neoadjuvant treatment patterns and surgery characteristics

In the FAS (n=30), most patients underwent two cycles of neoadjuvant therapy (46.67%), predominantly receiving nab-paclitaxel (66.67%) and nedaplatin (86.67%) as their taxane and platinum components, respectively (**Table 2**). Surgical resection was performed in 28 patients (93.33%), while one had not yet undergone surgery, and another had missing surgical data. The interval from the last neoadjuvant dose to surgery was 3–6 weeks in 14 (46.67%) patients and more than 8 weeks in 6 (20.00%). Among them, the delays were attributable to adverse event recovery (n=3), preoperative imaging reassessment (n=2), and patient preference (n=1), as shown in **Supplementary Figure S1**. The median interval from last dose to surgery was 6.22 weeks (range, 3.57-15.14). The median intraoperative blood loss was 100 mL (range, 50–300 mL). No significant differences in treatment or surgical parameters were observed between the MPR and non-MPR groups (all *P* > 0.05).

**Table 2:** Patterns of neoadjuvant treatment and surgical management.

Characteristic	Overall (N = 30)*	MPR (N = 16)	Non-MPR (N = 12)	p (MPR vs non-MPR)
Taxane regimen, n (%)				0.826
Nab-paclitaxel	20 (66.67)	10 (62.50)	9 (75.00)	
Paclitaxel	7 (23.33)	5 (31.25)	2 (16.67)	
Paclitaxel liposome	3 (10.00)	1 (6.25)	1 (8.33)	
Platinum regimen, n (%)				0.754
Cisplatin	3 (10.00)	1 (6.25)	2 (16.67)	
Carboplatin	1 (3.33)	1 (6.25)	0 (0.00)	
Nedaplatin	26 (86.67)	14 (87.50)	10 (83.33)	
Neoadjuvant cycles, n (%)				1.000
1	1 (3.33)	0 (0.00)	0 (0.00)	
2	14 (46.67)	8 (50.00)	5 (41.67)	
3	11 (36.67)	6 (37.50)	5 (41.67)	
>3	4 (13.33)	2 (12.50)	2 (16.67)	
Last dose to surgery (weeks), median (range)	6.22 (3.57,15.14)	6.00 (3.57,10.43)	6.86 (4.43,15.14)	0.427
Last dose to surgery interval (weeks), n (%)				0.691
3-6	14 (46.67)	9 (56.25)	5 (41.67)	
6-8	8 (26.67)	4 (25.00)	4 (33.33)	
>8	6 (20.00)	3 (18.75)	3 (25.00)	
Surgical resection performed*, n (%)	28 (93.3)	16 (100.0)	12 (100.0)	
Blood loss (mL), median(range)	100 (50,300)	100 (50,150)	150 (50,300)	0.077

*One patient has not yet undergone surgery, and one patient had missing surgical information.

### 3.3 Radiologic and pathological responses

All 30 patients had evaluable radiologic assessments. In the FAS (n=30), two patients (6.67%) achieved complete response (CR) and 22 (73.33%) achieved partial response (PR), with one patient experiencing disease progression (PD), resulting in an ORR of 80.00% (95% CI, 61.43–92.29%) and a DCR of 96.67% (95% CI, 82.78–99.92%) (**Table 3**). Notably, the proportion of radiologic responders was significantly higher in the MPR group than in the non-MPR group (*P* = 0.01).

**Table 3:** Radiologic responses.

Characteristic	Total (n = 30)*	MPR (n = 16)	non-MPR (n = 12)	*P* value
Best response				0.01
CR	2 (6.67)	2 (12.50)		
PR	22 (73.33)	14 (87.50)	7 (58.33)	
SD	5 (16.67)		4 (33.33)	
PD	1 (3.33)		1 (8.33)	
ORR (CR+PR)	24 (80.00)	16 (100.00)	7 (58.33)	
95% CI	61.43-92.29	79.41-100.00	27.67-84.83	
DCR(CR+PR+SD)	29(96.67)	16(100.00)	11(91.67)	
95% CI	82.78-99.92	79.41-100.00	61.52-99.79	

*One patient has not yet undergone surgery, and one patient had missing surgical information. ORR, objective response rate; DCR, disease control rate; CR, complete response; PR, partial response; SD, stable disease.

Pathological responses are shown in **Table 4**. Among 28 patients in the PPS set, MPR and pCR were achieved respectively in 16 (57.14%; 95% CI, 39.07–72.49%) and 8 (28.57%; 95% CI, 15.25–47.06%) patients, with R0 resection in 92.86%. Pathologic total downstaging was noted in 71.43% (95% CI, 52.94–84.75%), including T-stage downstaging in 89.29% and N-stage downstaging in 82.14%. The findings in sensitivity Set A were comparable to those observed in the PPS population, with similar MPR and pCR rates (57.14% [95% CI, 36.55–75.53%] and 28.57% [95% CI, 13.81–49.96%]), respectively, and a consistent frequency of pathologic downstaging (76.19%; 95% CI, 54.91-89.37%). Additionally, pathologic downstaging was more frequently observed in patients who achieved MPR than in those without MPR (PPS set, *P* = 0.044), and a similar trend was observed in the sensitivity Set A (*P* = 0.006, **Supplementary Figure S2A, B**).

**Table 4:** Pathological responses.

	PPS set		Sensitivity Set A	
Characteristic, n (%)	Total (N = 28)	95% CI	Total (N = 21)	95% CI
R0	26 (92.86)	77.35-98.02	20 (95.24)	77.33-99.15
MPR	16 (57.14)	39.07-73.49	12 (57.14)	36.55-75.53
PCR	8 (28.57)	15.25-47.06	6 (28.57)	13.81-49.96
Postoperative Downstaging, n (%)				
total downstaging	20 (71.43)	52.94-84.75	16 (76.19)	54.91-89.37
T downstaging	25 (89.29)	72.80-96.29	18 (85.71)	65.36-95.02
N downstaging	23 (82.14)	64.41-92.12	19 (90.48)	71.09-97.35

PPS, patients who underwent surgery with evaluable pathology (n = 28); Sensitivity Set A, stage II–III patients with surgery ≤8 weeks after neoadjuvant therapy (n = 21). MPR, major pathological response; PCR, pathological complete response.

### 3.4 Survival outcomes

As of the data cutoff on May 31, 2025, the median follow-up duration was 8.89 months. Owing to the short follow-up period and the limited number of events, both EFS and OS data were immature. In the FAS set of 30 patients, the preliminary median EFS was 29.97 months (95% CI, 20.33–NR), with only six EFS events (20.00%) observed (**Figure 1A**), and the 12-month EFS rate was 91.83% (95% CI, 81.41–100%). Only one death event in the overall cohort was due to pneumonia complicated by sepsis, rather than disease progression, with the estimated 24-month OS rate at 90.00% (95% CI: 73.20-100%) (**Figure 1B**). In supportive analyses, the median EFS was not reached in either the PPS set (n = 28; 95% CI, 14.70–NR) or the sensitivity analysis Set A (n = 21; 95% CI, 14.70–NR), as shown in **Supplementary Figures S3A** and **S3B**, respectively.

**Figure 1:**
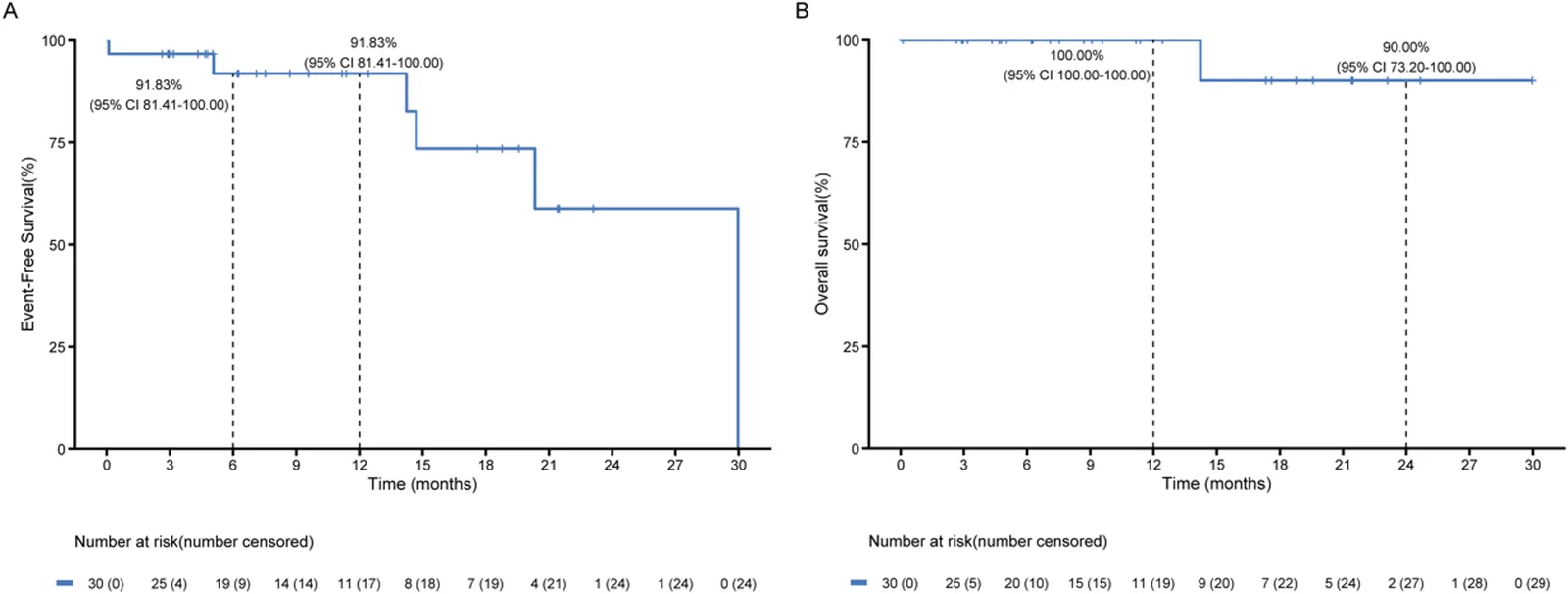
Kaplan–Meier estimates of event-free survival (EFS) and overall survival (OS) in the full analysis set (n = 30). **(A)** The median EFS was 29.97 months. The 6-month, 12-month, and 24-month EFS rates were 91.83%, 91.83%, and 75.00%, respectively. Tick marks indicate censored observations. **(B)** The median OS was not reached. The 12-month OS rate was 100.00%, and the 24-month OS rate was 90.00%. Tick marks indicate censored observations.

### 3.5 Safety

The safety profile of all 30 patients who received serplulimab-based neoadjuvant chemoimmunotherapy is summarized in **Table 5**. Overall, 12 patients (40.00%) experienced adverse events (AEs), most of which were grade 1–2 in severity (36.67%). No grade 4–5 AEs were observed. Immune-related adverse events (irAEs) were reported in 11 patients (36.7%), of which two (6.7%) were grade 3. The most common irAEs included hematological toxicities such as anemia, neutropenia, and leukopenia, as well as increased ALT and AST. All 28 patients who underwent surgery successfully completed resection, with no immune-related surgical complications or unexpected perioperative morbidity reported. All AEs resolved after appropriate treatment.

**Table 5:** Safety profile of patients.

	Total (n=30)	Grade 1–2	Grade 3
AEs	12 (40.00)	11 (36.67)	2 (6.67)
irAEs	11 (36.67)	10 (33.33)	2 (6.67)
Anemia	7 (23.33)	7 (23.33)	0
Decreased neutrophil count	5 (16.67)	5 (16.67)	0
Decreased white cell count	5 (16.67)	5 (16.67)	0
Increased ALT	4 (13.33)	4 (13.33)	0
Increased AST	4 (13.33)	4 (13.33)	0
Blood creatinine	3 (10.00)	3 (10.00)	0
Nausea	2 (6.67)	2 (6.67)	0
Maculopapular rash	2 (6.67)	2 (6.67)	0
Pruritus	2 (6.67)	2 (6.67)	0
Rash	2 (6.67)	2 (6.67)	0
Pyrexia	1 (3.33)	0	1 (3.33)
Vomiting	1 (3.33)	1 (3.33)	0
Pneumonia	1 (3.33)	0	1 (3.33)

AEs, adverse events; irAEs, immune-related adverse events; ALT, alanine aminotransferase; AST, aspartate aminotransferase; ALT, alanine aminotransferase.

### 3.6 Exploratory analyses

In the PPS population (n = 28), exploratory analyses were conducted to further evaluate pathological efficacy. Compared with the neoadjuvant chemotherapy control arms of the pivotal phase III trials KEYNOTE-671 and CheckMate-816, in which the MPR rates were 8.9% and 11.0%, respectively (**Figure 2**), the MPR rate in the present study reached 57.1% (90% CI, 43.3–100.0%) and was significantly higher than both historical reference values (both *P* < 0.001) (14, 15). A descriptive comparison of irAEs with these two trials is presented in **Supplementary Table 1**. The incidence of any-grade irAEs in our cohort (36.7%) was higher than that reported in KEYNOTE-671 (25.3%) and the estimated incidence in CheckMate-816 (~19.8%), whereas the incidence of Grade ≥3 irAEs (6.7%) was generally comparable (4.4%, ~3.4%).

**Figure 2:**
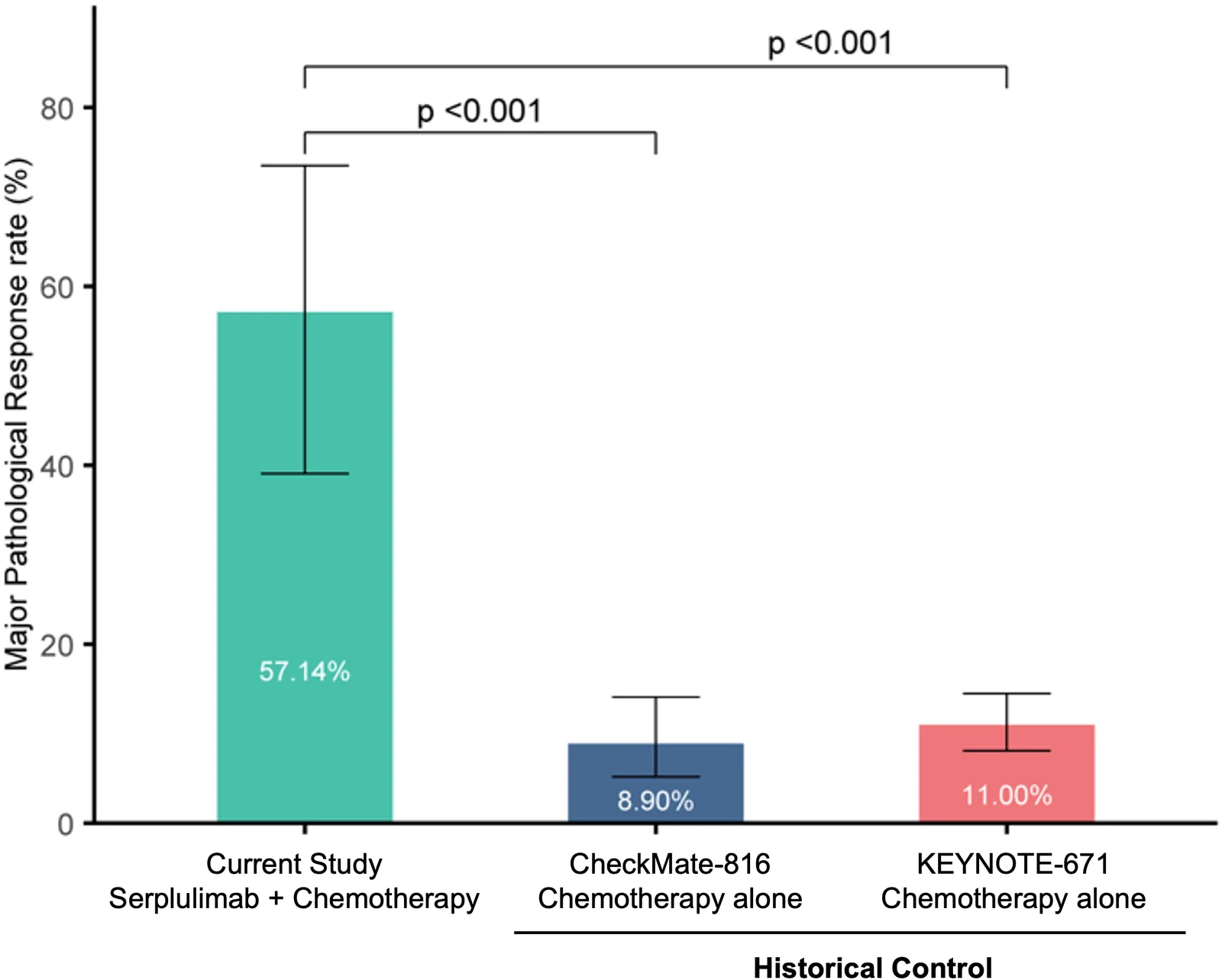
One-sample threshold test comparing the major pathological response (MPR) rate with the historical neoadjuvant chemotherapy group. Error bars represent 95% confidence intervals. *P* values were derived from exact one-sided one-sample binomial tests.

In both the PPS and sensitivity set A, univariate logistic regression suggested that clinical T stage and post-neoadjuvant NLR were significantly associated with MPR (**Table 6**). To identify independent predictors of MPR, a multivariate logistic regression model was constructed to simultaneously adjust for key clinical confounders. In the multivariate analysis, advanced clinical T stage (T3–T4) emerged as a strong independent predictor of a lower MPR rate in sensitivity set A (OR = 0.04, 95% CI 0.00–0.87, *P* = 0.040), while exhibiting a borderline significant trend in the PPS set (OR = 0.20, 95% CI 0.04–1.19, *P* = 0.078). Importantly, a low post-neoadjuvant NLR (<3) remained significantly and independently associated with a higher likelihood of achieving MPR, particularly within sensitivity set A (OR = 15.87, 95% CI 1.16–218.7, *P* = 0.039). The NLR cutoff of 3 was adopted based on prior literature demonstrating its prognostic significance in NSCLC (16). Conversely, other baseline clinical characteristics—including age, smoking history, comorbidities, PD-L1 expression, neoadjuvant cycles, and surgical intervals—showed no significant associations with MPR in either univariate or multivariate assessments (all *P* > 0.05).

**Table 6:** Univariate and multivariate regression analysis of clinical factors associated with MPR.

Characteristic	PPS set (n=28)				Sensitivity set A (n=21)			
	Univariate		Multivariate		Univariate		Multivariate	
	OR (95% CI)	p-value			OR (95% CI)	p-value		
Age (years)								
<65	-				-			
≥65	3.86 (0.80-22.80)	0.106			7.00 (1.10-64.69)	0.054		
Smoking history								
No	-				-			
Yes	1.20 (0.25-6.12)	0.820			1.00 (0.16-6.75)	1.000		
Comorbidity								
No	-				-			
Yes	0.43 (0.05-3.08)	0.401			0.32 (0.01-3.94)	0.384		
PD-L1 TPS,								
<1%	-				-			
1-49%	0.75 (0.08-7.25)	0.792			0.33 (0.01-4.26)	0.410		
≥50%	1.50 (0.12-35.30)	0.755			1.33 (0.10-32.55)	0.829		
Clinical T stage								
T1-T2	-		-		-		-	
T3-T4	0.17 (0.03-0.81)	**0.033**	0.20 (0.04-1.19)	0.078	0.07 (0.00-0.63)	**0.035**	0.04 (0.00,0.87)	**0.040**
Clinical N stage								
N-	-				-			
N+	0.64 (0.03-7.51)	0.726			-	-		
Clinical stage								
IB	-				-			
II	1.00 (0.00-Inf)	1.000			-			
III	0.00 (NR-Inf)	0.996			0.00 (NR-Inf)	0.994		
Neoadjuvant cycles								
2	-				-			
3	0.75 (0.14-3.88)	0.729			1.11 (0.16-8.07)	0.914		
>3	0.63 (0.06-6.63)	0.683			0.33 (0.01-4.68)	0.427		
Last dose to surgery interval (weeks)								
>8	-				-			
6-8	1.00 (0.11-8.76)	1.000			-			
≤6	1.8 (0.25-13.44)	0.552			1.60 (0.27-10.01)	0.605		
Pre-neoadjuvant NLR								
≥3					-			
<3	2.33 (0.52-11.44)	0.278			4.00 (0.68-28.61)	0.138		
Post-neoadjuvant NLR								
≥3	-		-		-		-	
<3	6.00 (1.23-35.19)	**0.033**	4.88 (0.84-28.37)	0.078	10.00 (1.47-100.80)	**0.028**	15.87 (1.16-218.07)	**0.039**

OR, odds ratio; CI, confidence interval. Reference indicates the comparison group (OR = 1.00). PPS, patients who underwent surgery with evaluable pathology (n = 28); Sensitivity Set A, stage II–III patients with surgery ≤8 weeks after neoadjuvant therapy (n = 21). Bold values indicate statistically significant differences (P < 0.05).

## 4 Discussion

Landmark clinical trials have established perioperative immunochemotherapy as a practical approach for resectable NSCLC. The CheckMate-816 demonstrated that adding nivolumab to platinum-based chemotherapy in the neoadjuvant setting improved the MPR rate from 8.9% to 36.9%, significantly prolonged EFS compared with chemotherapy alone (43.8 vs. 18.4 months; HR [95% CI]: 0.66 [0.49–0.90]), and increased the 4-year OS rate by 13% (71% vs. 58%) (15, 17). The KEYNOTE-671 trial further advanced the field. Perioperative pembrolizumab combined with platinum-based chemotherapy increased the MPR rate from 11.0% to 30.2%, the pCR rate from 4.0% to 18.1%, and extended the median EFS to 47.2 months (vs. 18.3 months for the control group; HR [95% CI]: 0.59 [0.48-0.72]). The addition of PD-1/PD-L1 blockade to platinum-based chemotherapy has been shown to markedly improve outcomes over chemotherapy alone (12). Although recent trials have expanded the evidence for perioperative immunotherapy, they have primarily focused on drug comparisons and have not clarified how neoadjuvant is implemented in real-world practice, particularly for driver gene–negative sq-NSCLC. Therefore, we undertook a multicenter real-world observational study to evaluate the efficacy and safety and to objectively describe the treatment patterns, including regimen use, cycle variability, and surgical timing of neoadjuvant serplulimab with platinum–taxane chemotherapy to explore the feasibility in a real-world setting.

Of the 30 patients included in the analysis, most patients received two to three cycles of serplulimab-based chemoimmunotherapy, and 80.0% achieved a radiologic response. Since one patient has not yet undergone surgery and another patient had missing surgical information, 28 patients had surgical pathology results. Among them, half underwent resection within 6 weeks after neoadjuvant therapy, while 20% had a surgery interval longer than 8 weeks. Regarding surgical results, most patients had R0 resection (92.86%) and achieved MPR and pCR rates of 57.14% and 28.57%, respectively. Pathologic downstaging was observed in more than 70% of cases, with T- and N-stage downstaging rates exceeding 80%. To further contextualize these findings, a one-sample threshold test was performed to compare the observed MPR rate with historical benchmarks from the neoadjuvant chemotherapy group (8.9% and 11.0%) (14, 15). The MPR of 57.1% (90% CI, 43.3–100.0%) observed in this study was significantly higher than both reference values (*P* < 0.001), suggesting a potential pathological advantage of serplulimab-based immunochemotherapy over chemotherapy alone. However, our survival data—including both OS and EFS—remain highly immature due to the short follow-up and the occurrence of only six EFS events and one death. Therefore, these findings must be considered purely exploratory, yet they still hold great potential to translate into favorable long-term survival outcomes. Regarding safety, the treatment demonstrated a manageable safety profile, with grade 3 AEs occurring in 2 patients (6.7%), all of which resolved with supportive care. The higher any-grade irAE rate observed in our cohort (36.7%) compared with landmark trials should be interpreted with caution. While KEYNOTE-671 formally reported an overall irAE rate, the corresponding rate for CheckMate-816 was estimated from the published frequencies of individual immune-mediated adverse events, precluding a fully consistent cross-trial comparison. Moreover, the higher rate observed in our study may partly reflect the more comprehensive AE capture inherent to real-world clinical practice. Recently, the prospective ECTOP-1013 phase II trial investigated a simplified perioperative regimen of serplulimab plus taxane–carboplatin in 30 patients with resectable stage II–IIIA sq-NSCLC, reporting MPR and pCR rates of 76.67% and 50.00%, respectively (18). Compared with ECTOP-1013, our real-world cohort included a higher proportion of stage III disease (86.7% vs. 53.33%), which may partly explain the relatively lower MPR and pCR rates observed in our study. Despite differences in study design and patient characteristics, the consistent demonstration of promising pathological responses across both a prospective trial and a real-world setting provides mutually supportive evidence for the efficacy of neoadjuvant serplulimab-based chemoimmunotherapy in sq-NSCLC.

The focus on driver gene–negative sq-NSCLC is particularly relevant because this population represents the biologically and clinically ideal target for perioperative immunotherapy. Compared with lung adenocarcinoma, sq-NSCLC is more closely linked to smoking and mostly occurs in male patients. This pattern was clear in our cohort, where 29 out of 30 patients were male. Furthermore, sq-NSCLC rarely harbors targetable driver mutations such as EGFR, ALK, or ROS1 rearrangements. Instead, it is characterized by higher genomic instability, which broadens the range of neoantigens and could improve tumor immunogenicity (19). Consistent with this biology, emerging evidence indicated that sq-NSCLC, characterized by elevated tumor mutational burden (TMB), higher PD-L1 expression, greater infiltration of functional tumor-infiltrating lymphocytes (TILs), and a chemokine-rich, immune-inflamed microenvironment, may derive greater benefit from immune checkpoint blockade than adenocarcinoma (20–23). In addition, a genome-wide analysis also showed that tumors with higher mutational or neoantigen burden and an inflamed immune milieu are more likely to respond to checkpoint inhibitors (24). Clinical validation of these findings was exemplified by the phase III ASTRUM-004 trial, which demonstrated that serplulimab significantly improved survival compared with chemotherapy in advanced sq-NSCLC (median OS 22.7 vs 18.2, HR 0.73; median PFS 8.3 vs 5.7 months; HR 0.53) (13). However, dedicated studies investigating immunotherapy in early-stage sq-NSCLC, especially in the perioperative setting, remain limited. Our findings indicate that serplulimab may also have therapeutic potential as a neoadjuvant agent, underscoring this population as a priority for future prospective evaluation.

To assess the robustness of surgical outcomes, we conducted a sensitivity analysis (sensitivity set A, n = 21) that included only patients with clinical stage II–III disease and a surgery interval ≤8 weeks. This approach minimized potential heterogeneity introduced by early-stage tumors, which typically have lower tumor burden and inherently better prognosis, potentially inflating pathological response or survival estimates. In addition, previous studies have suggested that the time from last neoadjuvant treatment to surgery (TTS) may influence pathological response and long-term outcomes. For instance, a real-world retrospective study showed that TTS >6 weeks was associated with shorter survival (median OS: 44.1–36.1 months vs 60.7 months for ≤6 weeks) and a trend toward higher 90-day postoperative mortality (25), suggesting that prolonged TTS may negatively impact pathological and survival outcomes. In real-world practice, however, keeping TTS within the 6-week window can be challenging due to patient recovery, perioperative assessment, and healthcare resources allocation. In our study, half of the patients underwent surgery only after 6 weeks; therefore, we defined TTS ≤8 weeks as a pragmatic boundary for the sensitivity analysis. Notably, the MPR and pCR trends in sensitivity set A remained consistent with those observed in the PPS. This result minimizes potential bias from excessive surgical delays and more accurately represents the real-world objective efficacy of serplulimab neoadjuvant therapy.

In our study, radiologic response after neoadjuvant immunochemotherapy showed a significant association with pathological response, as most radiologic responders achieved MPR in resected specimens (*P* = 0.01). Similar findings were reported in prior studies (26, 27). However, this correlation was not absolute; seven patients who achieved PR radiologically did not attain MPR, underscoring the inherent limitations of RECIST in fully reflecting pathological remission. Volumetric CT has been shown to provide a more sensitive and comprehensive assessment of tumor burden and to offer superior predictive value for MPR and pCR compared with unidimensional RECIST (28). Nevertheless, its broader implementation in routine practice is restricted by the requirement for specialized software, additional radiologist workload, and higher analytical costs. Given these practical constraints, RECIST remains a feasible and reasonably reliable method for response evaluation in real-world settings, despite its imperfect concordance with pathological outcomes.

Besides, to identify factors associated with pathological response, we performed an exploratory univariable and multivariate analysis. Advanced clinical T stage (T3–T4) and a low post-neoadjuvant NLR ratio (NLR < 3) emerged as independent factors of MPR in sensitivity set A. Patients with higher T clinical stage were substantially less likely to achieve MPR, which may reflect the more immunosuppressive tumor microenvironment often observed in larger or more invasive tumors, including hypoxia, stromal fibrosis, and decreased effector T-cell infiltration, all of which can limit immune-mediated tumor response clearance (29–32). Several retrospective and real-world analyses have also suggested that larger tumor size or more advanced T stage may be associated with poorer pathological response or worse prognosis after neoadjuvant therapy (33). Conversely, a low post-treatment NLR was associated with a markedly higher likelihood of MPR in our cohort. This finding aligns with multiple studies and meta-analyses showing that higher NLR, reflecting systemic inflammation and relative lymphopenia, is linked to lower response rates to immune checkpoint inhibitors in NSCLC, whereas a lower NLR correlates with improved response and prognosis (34, 35). Importantly, the clinical utility of NLR is particularly relevant in real-world settings. As a readily available, low-cost, and routinely measured biomarker, NLR requires no specialized assays, is easily incorporated into standard follow-up, and can be repeatedly assessed throughout the treatment course. These advantages make NLR a practical tool for early treatment evaluation and risk stratification in everyday clinical practice, providing timely insights into therapeutic efficacy and allowing clinicians to adjust management strategies accordingly.

However, several limitations must be acknowledged. The sample size was small, which increases the risk of potential selection bias and unmeasured confounding. The follow-up duration was short, and there were few survival events, which limits the ability to interpret long-term efficacy. Additionally, as a single-arm observational study, no concurrent chemotherapy comparator cohort was available. Although the chemotherapy arms of CheckMate-816 and KEYNOTE-671 were used as historical benchmarks, differences in baseline characteristics between these historical cohorts and our study population cannot be fully accounted for in a cross-trial comparison, introducing potential selection and temporal biases. Therefore, the results of the historical comparison and one-sample threshold test should be interpreted cautiously and considered exploratory. Additionally, certain baseline variables, including BMI and COPD status, were not routinely documented in the electronic medical records and could not be retrieved for analysis. Future prospective studies with larger cohorts and longer follow-up are needed to confirm these findings and explore mechanisms underlying response.

## 5 Conclusion

In conclusion, neoadjuvant serplulimab combined with chemotherapy demonstrated substantial pathological response rates, favorable surgical outcomes, and acceptable safety in patients with locally advanced, resectable sq-NSCLC in a real-world setting. These findings provide supportive evidence for the clinical application of serplulimab in the perioperative management of sq-NSCLC and warrant further validation in larger, controlled studies.

## Data Availability

The original contributions presented in the study are included in the article/**Supplementary Material**. Further inquiries can be directed to the corresponding author/s.
