# Supplementary material for: Neoadjuvant serplulimab combined with chemotherapy in locally advanced driver gene-negative squamous NSCLC: a multicenter observational study
Source: Front Immunol. 2026 Sep 24;17:1882282. doi: 10.3389/fimmu.2026.1882282 (PMC13646145; doi:10.3389/fimmu.2026.1882282)
Supplement: Supplementary file 1 [file SupplementaryFile1.docx]

Supplementary Material

# 1 Supplementary Figures

**Figure S1. Patient selection and exclusion flowchart for the supportive analyses.**

Sq-NSCLC, squamous non–small cell lung cancer; PPS, pathological assessment set; AE, adverse event.

**Figure S2. Association between major pathologic response and pathologic downstaging.** (A) Association in the per-protocol set. (B) Association in the sensitivity analysis set A.

**Figure S3. Event-free survival in the supportive analysis sets.** (A) The per-protocol set. (B) The sensitivity analysis set A.

# 2 Supplementary Table

**Table S1. Comparison of safety profiles between the current study and the KEYNOTE-671 and CheckMate-816 trials.**

|  | Current Study | KEYNOTE-671 | CheckMate-816 |
| --- | --- | --- | --- |
| Any grade irAE, % | 36.7% | 25.3% | ~ 19.8%^†^ |
| Grade ≥3 irAE, % | 6.7% | 5.8% | ~ 3.4%^†^ |

AE, adverse event; irAE, immune-related adverse event. † The irAE rate not formally reported; estimated from individual immune-mediated AE events
